# INFLUENCES OF CAREWORK INTENSITY ON DAILY TIME USE PATTERNS OF CAREGIVERS

**DOI:** 10.1093/geroni/igz038.2965

**Published:** 2019-11-08

**Authors:** Liana C Sayer

**Affiliations:** University of Maryland Department of Sociology, College Park, Maryland, United States

## Abstract

With the aging of the Baby Boom generation, increasing numbers of older adults require assistance in their daily lives and most help comes from family members. Delays in childbearing mean many adult elder care providers are simultaneously raising children. Although past research has documented disparities in psychological distress and financial costs, less is known about the social costs resulting from elder caregiving and how this varies by parental status. We examine the social costs of elder caregiving by comparing elder and child care configurations to investigate three questions. First, do the daily time use patterns of elder caregivers differ by parental status? Second, do the daily time use patterns of elder caregivers differ by caregiving intensity? Third, does caregiving intensity moderate associations of elder caregiving and parental status on daily time use? We address these questions using nationally representative time diary data from the 2011-2017 American Time Use Survey (ATUS).

